# Supplementary material for: Giant Viruses as a Source of Novel Enzymes for Biotechnological Application
Source: Pathogens. 2022 Dec 1;11(12):1453. doi: 10.3390/pathogens11121453 (PMC9787589; doi:10.3390/pathogens11121453)
Supplement: Supplementary file 1 [file pathogens-11-01453-s001.zip › Table S2.pdf]

Table S2. TM-scores of *B. cereus*, Tupanvirus and PBCV-1 target sequences compared to their templates from SWISS-MODEL and/or RoseTTAFold

	**SWISS-MODEL**			**RoseTTAFold**			
**Template/Target**	PDB ID: 6IGY	PDB ID: 5GQB	PDB ID: 6BT9	PDB ID: 6IGY	PDB ID: 5GQB	PDB ID: 6BT9	RoseTTA/ SWISS
B. cereus	0.1324	0.1418	**0.656**	0.1328	0.14	**0.63**	0.9893
**Tupanvirus**	0.1501	**0.1978**	0.168	0.1357	**0.1861**	0.181	0.7283
**PBCV-1**	**0.3295**	0.1557	0.1805	**0.2568**	0.1649	0.183	0.9034

6IGY: chitinase B *Aspergillus niger*; 5GQB: chitinase H *Ostrinia furnacalis*; 6BT9: chitinase *Bacillus thuringiensis*; TM-scores > 0.17: not randomly chosen unrelated proteins; TM-scores > 0.5: in about the same fold in protein structures. Selected best hit (scored) models/templates were highlighted in bold.
