# Supplementary figures and images for: Giant Viruses as a Source of Novel Enzymes for Biotechnological Application
Source: Pathogens. 2022 Dec 1;11(12):1453. doi: 10.3390/pathogens11121453 (PMC9787589; doi:10.3390/pathogens11121453)

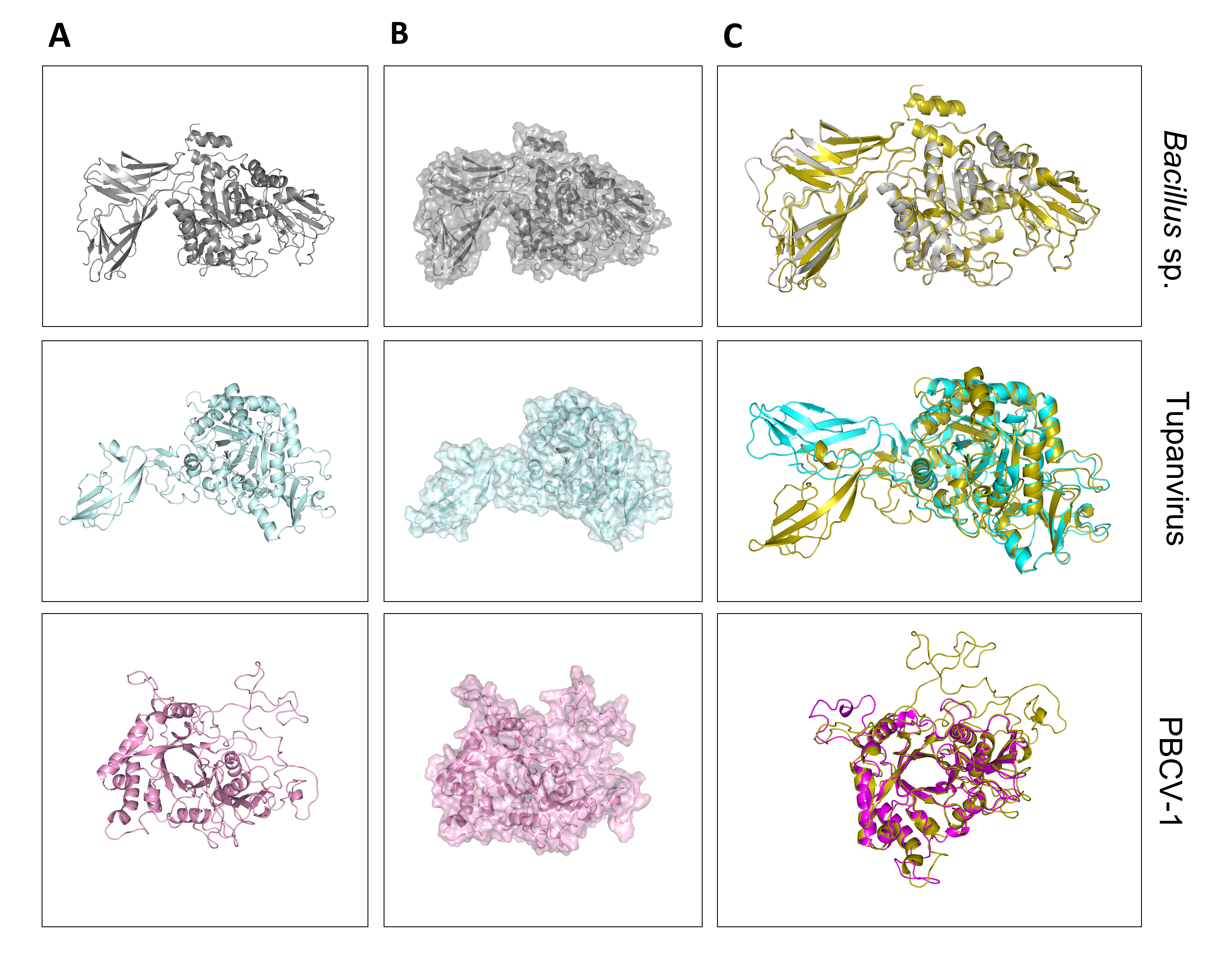

Supplement: Supplementary file 1 [file pathogens-11-01453-s001.zip › Figure_S1.tif]
